# Combined Effect of Light and Temperature on the Production of Saxitoxins in *Cylindrospermopsis raciborskii* Strains

**DOI:** 10.3390/toxins11010038

**Published:** 2019-01-14

**Authors:** Marcella C. B. Mesquita, Miquel Lürling, Fabiane Dorr, Ernani Pinto, Marcelo M. Marinho

**Affiliations:** 1 Laboratory of Ecology and Physiology of Phytoplankton, Department of Plant Biology, Rio de Janeiro State University, Rio de Janeiro 20550-900, Brazil; manzi.uerj@gmail.com; 2 Department of Environmental Sciences, Aquatic Ecology and Water Quality Management Group, Wageningen University, P.O. Box 47, 6700 AA Wageningen, The Netherlands; miquel.lurling@wur.nl; 3 Department of Aquatic Ecology, Netherlands Institute of Ecology (NIOO-KNAW), P.O. Box 50, 6700 AB Wageningen, The Netherlands; 4 Department of Clinical and Toxicological Analyses, School of Pharmaceutical Sciences, University of São Paulo, 05508000 São Paulo, Brazil; fabidorr@usp.br (F.D.); ernani@usp.br (E.P.)

**Keywords:** cyanobacteria, cyanotoxins, saxitoxins, intraspecific variability

## Abstract

*Cylindrospermopsis raciborskii* is a potentially toxic freshwater cyanobacterium that can tolerate a wide range of light and temperature. Due to climatic changes, the interaction between light and temperature is studied in aquatic systems, but no study has addressed the effect of both variables on the saxitoxins production. This study evaluated the combined effect of light and temperature on saxitoxins production and cellular quota in *C. raciborskii*. Experiments were performed with three *C. raciborskii* strains in batch cultures under six light intensities (10, 40, 60, 100, 150, and 500 μmol of photons m^−2^ s^−1^) and four temperatures (15, 20, 25, and 30 °C). The growth of *C. raciborskii* strains was limited at lower temperatures and the maximum growth rates were obtained under higher light combined with temperatures equal or above 20 °C, depending on the strain. In general, growth was highest at 30 °C at the lower light intensities and equally high at 25 °C and 30 °C under higher light. Highest saxitoxins concentration and cell-quota occurred at 25 °C under high light intensities, but were much lower at 30 °C. Hence, increased temperatures combined with sufficient light will lead to higher *C. raciborskii* biomass, but blooms could become less toxic in tropical regions.

## 1. Introduction

*Cylindrospermopsis raciborskii* is a freshwater cyanobacterium, which is widely distributed in tropical, subtropical, and temperate regions [1,2]. It can tolerate a wide range of temperatures and light intensities [3,4,5] and is able to produce distinct cyanotoxins, like cylindrospermopsins (CYN) and saxitoxins (STXs) [6]. The production of these cyanotoxins by *C. raciborskii* is related to its geographical distribution, where Brazilian strains produce STX derivatives [7,8,9] and Australian strains can produce CYNs [1,10], while North American strains are still uncertain about the production of this toxin [11,12], and European strains produce other, yet undescribed, neurotoxins [1].

The most common STXs are generally grouped according to their structural differences in the three main groups, namely the carbamoyl, dicarbamoyl, and sulfocarbamoyl derivatives [6]. The carbamoyl group (STXs) includes STX, neosaxitoxin (Neo) and the gonyatoxins (GTX 1–4), with STX the more studied [7,10,13,14]. The biosynthesis of STXs and the evolution of the genes responsible for their complex metabolism seem to indicate that these toxins are probably linked with ecological advantages for their producers [15,16,17,18]. Environmental factors that have been found upregulating STX biosynthesis are high light intensity [7], high and suboptimal temperature, extracellular salt (NaCl) [19], conductivity, and dissolved inorganic nitrogen (DIN) [20], whereas high nitrogen concentrations and darkness downregulated STX biosynthesis [7,19].

In Brazil, higher *C. raciborskii* densities and higher STX production were associated with lower temperatures in the field [21]. In contrast, few laboratory studies with isolated strains have observed increased production of STXs from low to high temperatures [22,23]. In addition to the effect of temperature, light intensity also affects the production of STXs: High light intensity (100 μmol of photons m^−2^ s^−1^) promotes the increase in production of STXs [7].

The number of studies combining light and temperature in *C. raciborskii* has amplified [3,4,24]. In general, combined high light intensities at high temperatures yielded increased growth rates in *C. raciborskii* strains [3,4]. However, the combined effect of light and temperature on STX production has not been studied. Understanding how the interaction of these two variables may affect the production of STXs is crucial for the management of toxic *C. raciborskii* blooms. In this study, we tested the hypothesis that (1) *C. raciborskii* strains would be able to grow in a wide range of temperatures, and (2) combined high light intensity and high temperature would increase the production of STXs. To this end, we evaluated the combined effects of light and temperature on saxitoxins production in three *C. raciborskii* strains isolated from a naturally eutrophic reservoir.

## 2. Results

### 2.1. Growth Rates

**Figure 1:**
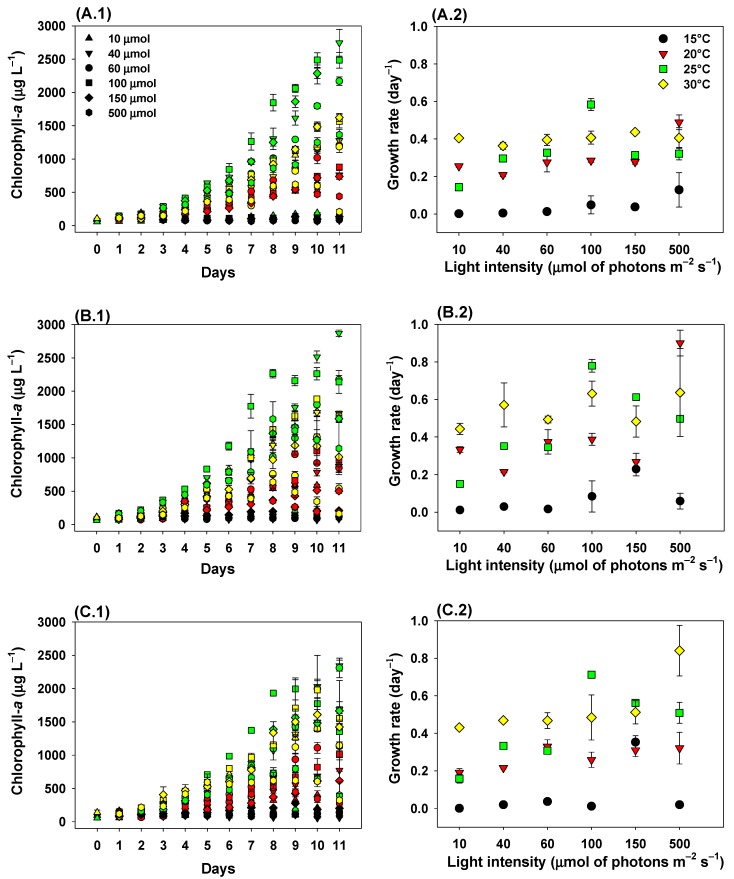
Growth based on chlorophyll-*a* (1) and calculated growth rates (2) of *Cylindrospermopsis raciborskii* strains under the combinations of six light intensities and four water temperatures. Letters indicated *C. raciborskii* strains: CYLCAM-01 (**A**); CYLCAM-02 (**B**) and CYLCAM-03 (**C**). Symbols indicate light intensities: Up triangles = 10 µmol photons m^−2^ s^−1^; down triangles = 40 µmol photons m^−2^ s^−1^; circles = 60 µmol photons m^−2^ s^−1^; squares = 100 µmol photons m^−2^ s^−1^; diamonds = 150 µmol photons m^−2^ s^−1^; hexagons = 500 µmol photons m^−2^ s^−1^; Colors indicate temperatures: black = 15 °C; red = 20 °C; green = 25 °C; yellow = 30 °C. Bars are standard deviations (n = 3).

Light, temperature, and light × temperature interaction had significant effects on growth rates of *C. raciborskii* strains (Table 1). All strains showed the lowest growth rates (*p* < 0.05) at 15 °C, combined with low light intensities (≤60 μmol of photons m^−2^ s^−1^) (Figure 1).

The increase in temperature was an important factor for the growth rate of CYLCAM-01 strain when combined with light intensities of 10 and 100 µmol photons m^−2^ s^−1^. CYLCAM-01 strain exhibited the highest growth rates (*p* < 0.05) at 25 °C when combined with 100 µmol photons m^−2^ s^−1^, reaching 0.58 ± 0.03 day^−1^ (Figure 1A1,A2). At light intensities of 40, 100, and 500 µmol photons m^−2^ s^−1^, the increase in temperature was a significant factor for the growth of CYLCAM-02 (Figure 1B1,B2). This strain showed the highest growth rate of the study (0.90 ± 0.06 day^−1^) when combining 500 µmol photons m^−2^ s^−1^ at 20 °C (*p* < 0.05). This strain exhibited an increase in the growth rate with increasing light intensity of up to 100 μmol photons m^−2^ s^−1^ at 25 °C, and reduction occurred when combined with the highest light intensities (*p* < 0.05). At 30 °C, the increase in light intensity did not affect the growth rate (*p* > 0.05) (Figure 1B2). CYLCAM-03 showed a significant increase in growth rate in 150 μmol of photons m^−2^ s^−1^ at 15 °C (Figure 1C1,C2). Likewise observed for CYLCAM-02, the increase in temperature was an important factor for the growth rate of CYLCAM-03 strain when combined with light intensities of 40, 100 and 500 µmol photons m^−2^ s^−1^. At 30 °C, only CYLCAM-03 strain showed a significant difference in growth rates, with an increase when combined with 500 μmol of photons m^−2^ s^−1^, reaching its highest growth rate (0.84 ± 0.13 day^−1^, *p* < 0.005).

### 2.2. Saxitoxins (STXs) Concentrations

**Figure 2:**
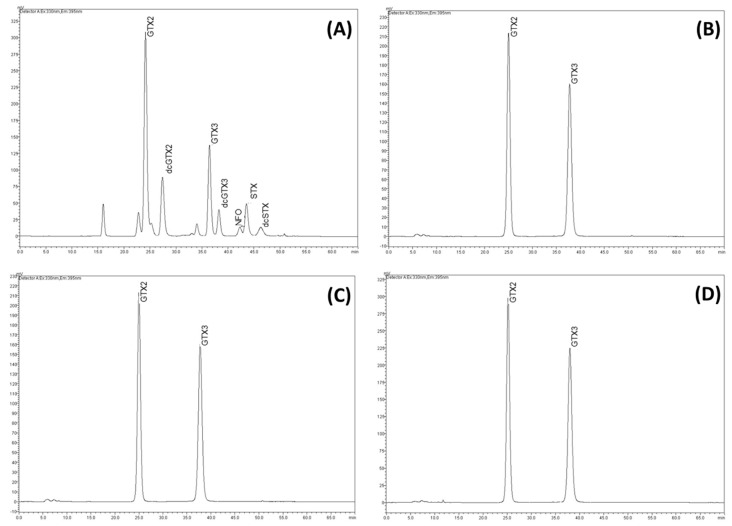
Typical HPLC-FD (HILIC) chromatograms of STX variants in *C. raciborskii* CYLCAM strains. Commercial standards of saxitoxins (**A**); *C. raciborskii* CYLCAM-01 (**B**); *C. raciborskii* CYLCAM-02 (**C**); *C. raciborskii* CYLCAM-03 (**D**). Chromatograms were acquired as described in Material and Methods section.

Typical chromatograms of CYLCAM-01, 02, 03 strains, as well as PSP analytical standards, can be seen in Figure 2, where we only found gonyatoxins (GTX-2 and GTX-3) (Figure 2). The saxitoxins concentration was made by the sum of GTX-2 and GTX-3 (Figure 2). Although some variation occurred, on average, each variant represented 50% of the total STXs (Figure S1, Supplementary Materials).

**Figure 3:**
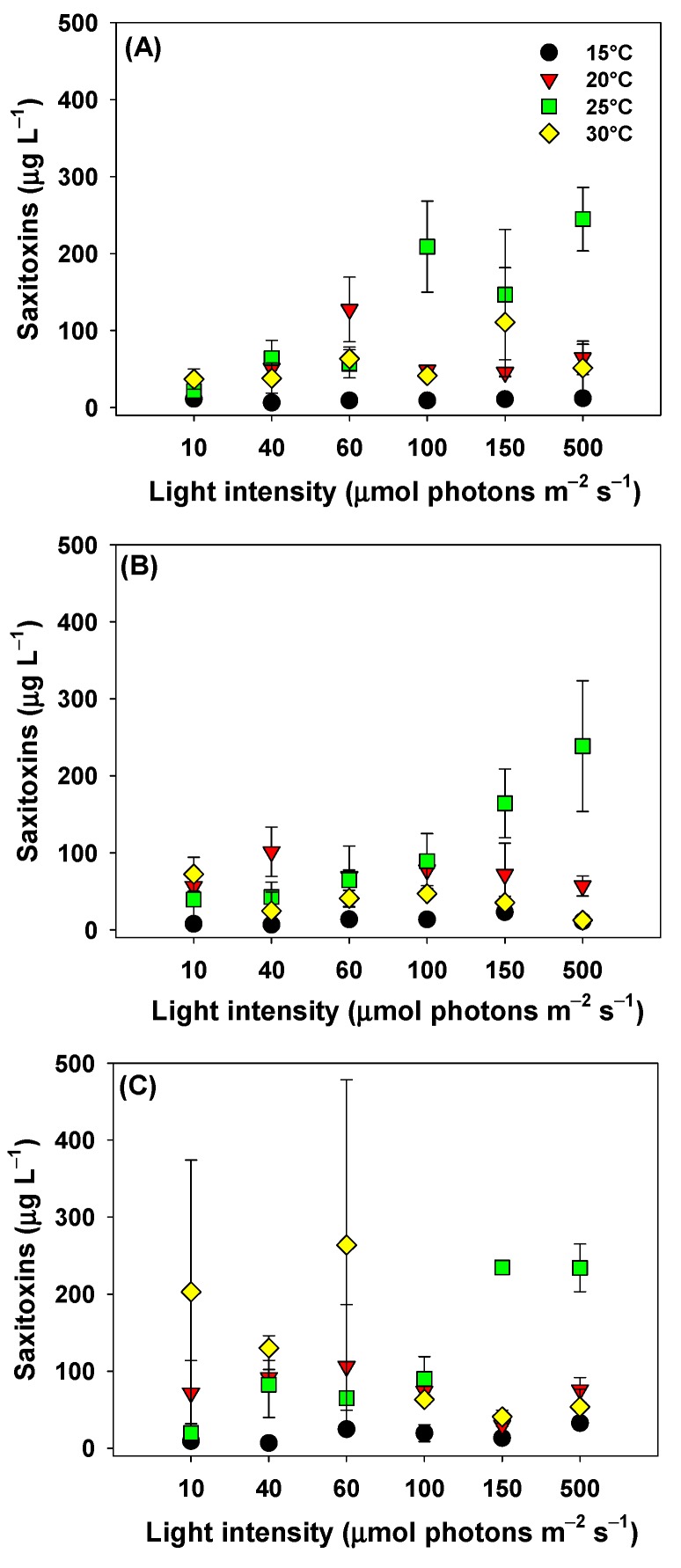
Production of STXs by three strains of *C*. *raciborskii* under the combinations of the six light intensities and four water temperatures. CYLCAM-01 (**A**); CYLCAM-02 (**B**) and CYLCAM-03 (**C**). Black circles = 15 °C; down red triangles = 20 °C; green squares = 25 °C; yellow diamonds = 30 °C. Bars on the vertical lines are standard deviations (n = 3).

Two-way ANOVA revealed a significant temperature and light intensity effect, and a significant interaction between the two factors on STXs concentrations in the cultures (Table 2). The three strains of *C. raciborskii* showed the lowest STXs concentrations (*p* < 0.05) at 15 °C, regardless of light intensity (Figure 3). The higher STXs values were recorded at 25 °C under high light (≥100 μmol of photons m^−2^ s^−1^) for all strains and at 30 °C for CYLCAM-03 in combination with low light (≤60 μmol of photons m^−2^ s^−1^) (Figure 3C) (*p* < 0.05).

The CYLCAM-01 strain showed no significant difference in STX production, regardless of tested temperature when combined with low light intensities (10 and 40 μmol of photons m^−2^ s^−1^) (*p* > 0.05). However, there was an increase of STXs when combining high light intensities (≥100 μmol of photons m^−2^ s^−1^) at 25 °C (Figure 3A) (*p* < 0.05). The CYLCAM-02 strain when cultivated at 10, 60 and 100 μmol of photons m^−2^ s^−1^ showed no significant difference in STXs production, regardless of the combined temperature (Figure 3B) (*p* > 0.05). This strain also did not show differences in STX production when submitted to 15, 20 and 30 °C, regardless of the combined light intensity (*p* > 0.05).

CYLCAM-03, when combined with 10 and 100 μmol of photons m^−2^ s^−1^, showed no significant difference in STX production, regardless of the combined temperature (Figure 3C) (*p* > 0.05). At 25 °C in combination with light intensities ≥150 μmol of photons m^−2^ s^−1^ CYLCAM-03 showed an increase in STXs production, but a decrease was observed at 30 °C when combined with light intensities ≥100 μmol of photons m^−2^ s^−1^ at 30 °C (*p* < 0.05).

**Figure 4:**
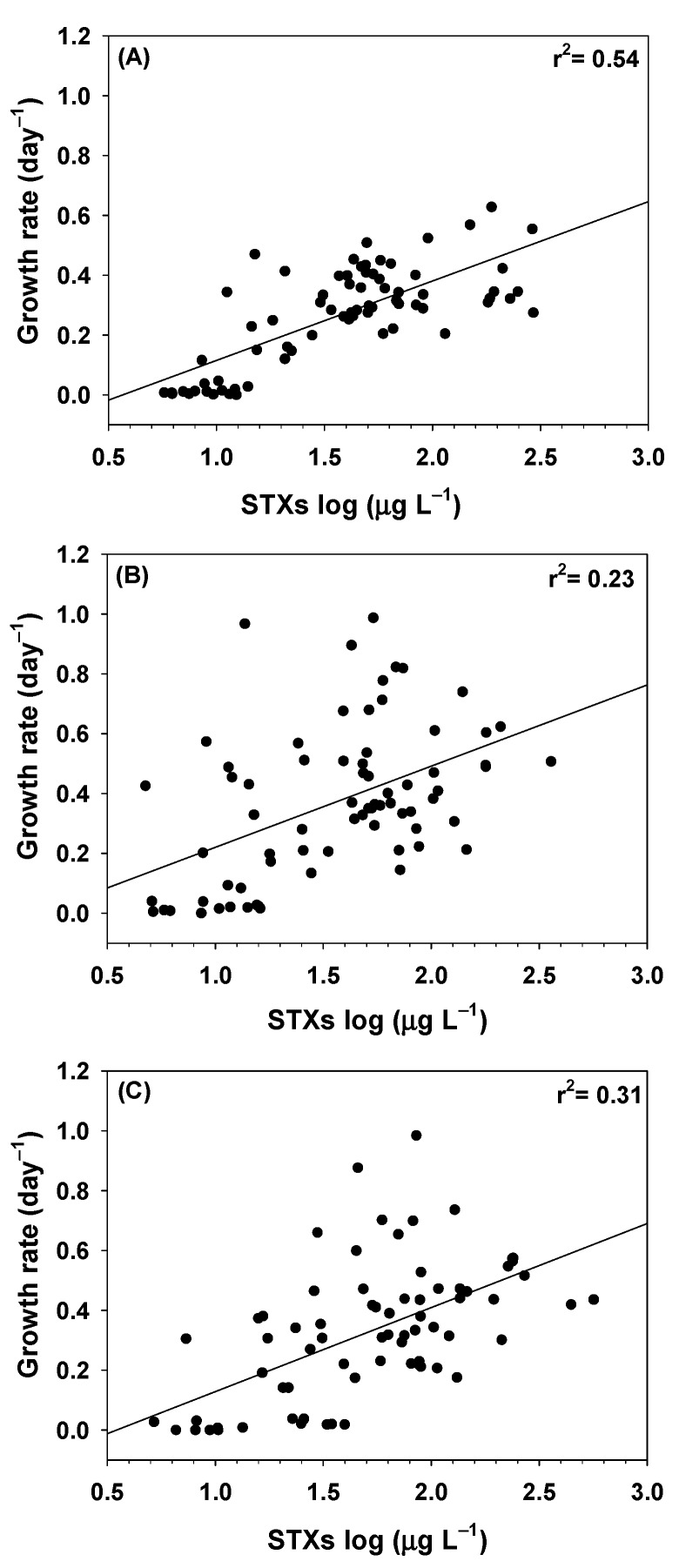
Relation between growth rate and STX production of three strains of *C. raciborskii* under the combinations of the six light intensities and four water temperatures. CYLCAM-01 (**A**); CYLCAM-02 (**B**) and CYLCAM-03 (**C**). Linear regression is indicated by black line.

Positive linear relations between growth rates and STX concentrations for the three *C. raciborskii* strains were found (Figure 4). CYLCAM-01 strain showed a high correlation (r^2^ = 0.54) (Figure 4A) in relation with others (Figure 4B,C).

### 2.3. STXs Cellular Quota

**Figure 5:**
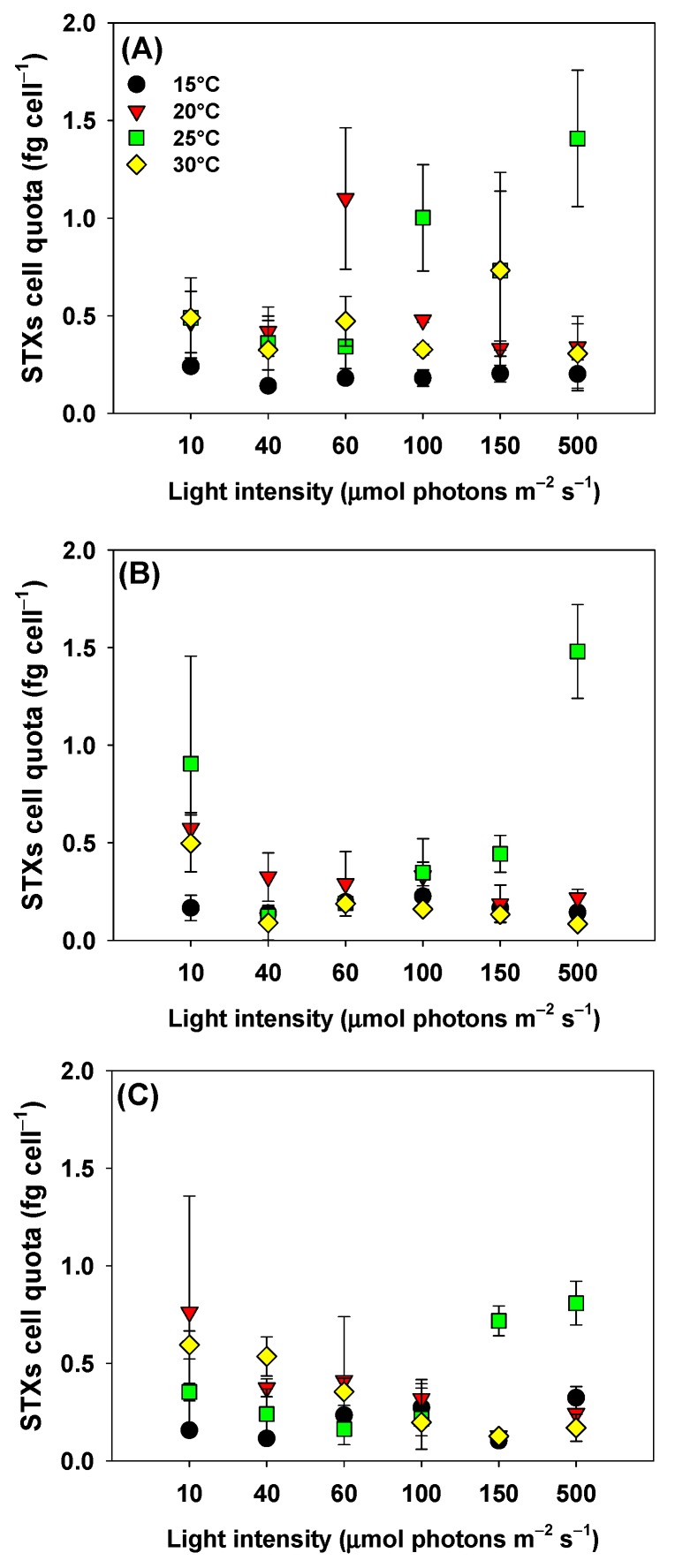
STXs cellular quota of three strains of *C*. *raciborskii* under the combinations of the six light intensities and four water temperatures. CYLCAM-01 (**A**); CYLCAM-02 (**B**) and CYLCAM-03 (**C**). Black circles = 15 °C; down red triangles = 20 °C; green squares = 25 °C; yellow diamonds = 30 °C. Bars on the vertical lines are standard deviations (n = 3).

Two-way ANOVA indicated no effect of light, temperature, or light × temperature interaction on STXs cellular quota of CYLCAM-03 (Table 3, Figure 5C). Conversely, CYLCAM-02 STXs cellular quotas were influenced by light, temperature or light × temperature interaction (Table 3). The highest STXs cellular quota was observed when combining 500 μmol of photons m^−2^ s^−1^ at 25 °C (Figure 5B) (*p* < 0.05). There was a reduction when this strain was combined with light intensities ≥40 μmol of photons m^−2^ s^−1^ at 30 °C (*p* < 0.05). CYLCAM-02 when combined at 15 °C and 20 °C did not exhibit a significant difference in the STXs cellular quota, regardless of tested light intensity (*p* > 0.05).

STXs cellular quota of CYLCAM-01 was not influenced by light, but significantly affected by temperature, and two-way ANOVA indicated a temperature × light interaction (Table 3). The CYLCAM-01 strain showed the highest STXs cellular quota when combined 60 μmol of photons m^−2^ s^−1^ at 20 °C and 100 and 500 μmol of photons m^−2^ s^−1^ at 25 °C (Figure 5A). When cultivated under light intensities of 10, 40, and 150 μmol of photons m^−2^ s^−1^, CYLCAM-01 did not show a significant difference in STXs cellular quota regardless of the combined temperature (*p* > 0.05). At the extreme temperatures tested (15 °C and 30 °C), the increase in light intensity did not resulted in significant difference of the STXs cellular quota (*p* < 0.05) (Figure 5A).

## 3. Discussion

In this study, we tested the hypothesis that *C. raciborskii* was able to grow in a wide range of temperatures and that *C. raciborskii* would increase the production of STXs when a high temperature was combined with high light intensity. Our results are in line with the first hypothesis, as strains expressed growth over a wide range of temperatures and when higher growth rate with increasing light intensity at temperatures equal to or greater than 20 °C was found. The highest STXs concentrations and cellular quota were not obtained under extreme conditions where both light and temperature were high. Hence, we reject our second hypothesis. In addition, our results showed that the strains exhibited different responses when submitted to different combinations of light and temperature tested for all parameters, reflecting intraspecific variability.

*C. raciborskii* can be considered a shadow species due to its low light requirement (Ik) [25], despite being a species of tropical origin, which global distribution is currently linked with increased temperature [1,2]. Most studies have shown that *C. raciborskii* strains require high light intensities to achieve maximum growth [3,4,26] and that optimum growth rates occur at a relatively elevated temperature (25–31 °C) [4,27]. In this study, the range of light intensity and temperature used encompassed those that have been reported for blooms of *C. raciborskii* in Brazil [28], respectively, 14 to 830 μmol photons m^−2^ s^−1^ of light [28] and high temperature, such as 27 °C [29]. Our results emphasized the ability of *C. raciborskii* to show optimal growth rates covering a wide range of light intensity and temperature.

Considering the effects of climatic changes in aquatic systems, the interaction between light and temperature on growth has been studied for *C. raciborskii* strains [3,4,5]. Controlled experiments revealed that higher light intensity (135 compared to 60 µmol photons m^−2^ s^−1^) and higher temperature (25 °C compared to 20 °C and 15 °C) promoted the growth of *C. raciborskii* [4]. Where, in the study of Bonilla et al. (2016) [4], no difference was found in the growth of *C. raciborskii* at 15 °C and 20 °C, in our study 15 °C clearly was the least favorable growth temperature. The *C. raciborskii* strains in this study demonstrated a great tolerance to grow over a wide range of light and temperature, where they obtained maximum growth rates in high light intensities combined with temperatures ≥20 °C. This is in agreement with *C. raciborskii* strains isolated from different regions of South America exhibiting maximum growth rates when high light intensities (≥90 μmol of photons m^−2^ s^−1^) were combined with high temperatures (25 °C and 31 °C) [3,4], regardless of the straight or spiral morphotype of the strains [3]. Although *C. raciborskii* evidently grows faster at higher light and higher temperatures, our study, as well as others (e.g., Bonilla et al. (2012) [5]; (2016) [4]; Bittencourt-Oliveira et al. (2012) [3]), show that *C. raciborskii* has the capacity to also grow under different combinations of light and temperature, emphasizing the physiological flexibility of *C. raciborskii,* which would be a favorable factor to explain the current distribution of this species worldwide.

*C. raciborskii* is a potential cyanotoxin-producing species [1,23]. One interesting aspect is that strains of this species from Oceania and Asia might produce cylindrospermospsins, while those in Brazil might produce saxitoxins, whereas also numerous strains there and elsewhere produce none of these toxins [1]. The biological reasons why Brazilian *C. raciborskii* strains produce STX variants instead of cylinsdrospermopsin are still not clear. However, based on genomic and target metabolomics approaches, Hoff-Risseti et al. (2013) [18] suggested that, besides the presence of *cyr* genes in Brazilian strains, some crucial steps are missing in the cluster, and only STX genes and variants were evidenced. The strains used in this study originated from Brazil and are STXs producers, which the production or/and increased concentration of this cyanotoxin has already been associated with some environmental factors like suboptimal temperature, light (intensity, quality, and dark cycles), high conductivity, dissolved inorganic nitrogen, and water hardness [7,13,20,21,22,23]. Clearly, the effects of individual environmental factors, such as (e.g., light and temperature) on STX concentrations have already been previously studied, but our study is the first paper that combined the effects of light and temperature in STXs concentrations in *C. raciborskii* strains. At 25 °C and in 150 and 500 µmol photons m^−2^ s^−1^, the highest STXs concentrations were measured in each strain.

Light and temperature are environmental variables that affect the photosynthetic apparatus and metabolism in phytoplankton species [26,30,31] and consequently affects the metabolic via to STX production [32]. Laboratory experiments demonstrate that STX production can be affected by light intensity. For example, *C. raciborskii* T3 strain showed greatest concentration of STX and NEO at 100 µmol photons m^−2^ s^−1^, compared with 50 and 150 µmol photons m^−2^ s^−1^ [7]. Our results showed the highest production of STXs at light intensities equal to or greater than 100 µmol photons m^−2^ s^−1^.

Besides light, also temperature may influence the STXs content in *C. raciborskii*, where, in general, higher temperatures yield higher STXs concentrations [22,23]. In our study, high amounts of STXs (GTX2 + GTX3) were found in the *C. raciborskii* strains reared at high light intensity at 25 °C. Interestingly, in our study, STX concentrations dropped sharply when *C. raciborskii* strains were cultured at 30 °C, compared with STX concentrations found in strains reared at 25 °C. This finding deviates from the increase in STX concentrations with temperatures up to 32 °C observed in another study [23]. However, where we detected GTX-2 and GTX-3, Rangel et al. (2016) [23] measured STX, dcSTX, NEO, and dcNEO in their strain. Although temperature can influence the composition of the STXs [22,23], as dcSTX was not detected in *C. raciborskii* strains at low temperature (17 °C and 19 °C), but it was present in cultures reared at 25 °C and 32 °C [22,23], no such shift in saxitoxin/gonyautoxin variants was measured in our study. Assuming that as a consequence of climate change the temperature will increases by 2–5 °C [33], the annual average in the tropical will be over 25 °C. This implies that, based on our results, strains and blooms of saxitoxin producing *C. raciborskii* might become less toxic under these future climate scenarios.

In addition to saxitoxin production, it is important to know how the behavior of *C. raciborskii* is in relation to STXs cellular quota, since there are few studies about it [21,23]. It was demonstrated with other cyanotoxins, such as cylindrospermopsins (CYN), that CYN cell quota of two *C. raciborskii* strains were not affected by the intensity of the surrounding light during growth [10], while another study with microcystin verified that an increase in temperature (18–30°C) can diminish the microcystin cell quota [34]. In relation with STXs cell quota, a field study observed the highest saxitoxin quota per trichome in the periods with the lowest *C. raciborskii* density and conclude that the production of this toxin or the selection of toxic strains may be an adaptation to the stress condition [21]. In laboratory experiments, the *C. raciborskii* strain LETC CYRF-01 increased toxicity, since the increase in saxitoxin production was related not only to the increase in density but to the amount of toxins per unit volume increased as well [23]. Our results of the STXs cellular quota were similar to those of STXs production, where *C. raciborskii* strains accumulated more intracellular STXs when they were combined with high light intensities at 25 °C and decreased by 30 °C. Therefore, our results contrast with idea that the STXs cellular quota is related to the less ideal conditions [21], since combinations of high light intensity at 25 °C promoted higher levels of STXs and high growth rate.

## 4. Conclusions

The growth of *C. raciborskii* strains isolated from a natural tropical eutrophic reservoir was limited by lower temperature in our laboratory experiments. The maximum growth rates were obtained in higher light intensity combined with temperatures equal to or above 20 °C, depending on the strain. Highest STX concentration and cell-quota occurred at 25 °C under high light intensities, but were much lower at 30 °C. Hence, increased temperatures combined with sufficient light will lead to higher *C. raciborskii* biomass, but blooms could become less toxic in tropical regions.

## 5. Material and Methods

Experiments were carried out with three strains of *Cylindrospermopsis raciborskii* (CYLCAM-01, CYLCAM-02 and CYLCAM-03). The strains were isolated between 2012 and 2013, from a tropical shallow reservoir located in a protected area (Pedra Branca State Park—22°55 54.44 S/43°28 22.44 W) in the western part of the municipality of Rio de Janeiro (Brazil). Currently the strains are maintained in the Culture Collection of the Laboratory of Ecology and Physiology of Phytoplankton, University of Rio de Janeiro State (UERJ) under the following conditions: WC medium [35]; temperature, 25 °C; irradiance, 30 µmol photons m^−2^ s^−1^ provided by daylight fluorescent lamps (Sylvania T10–20 watts–5000 k) and measured with a Li-COR quanta meter sensor; photoperiod, 12–12 h light-dark cycle. Cultures were not grown axenically, but regular microscopic inspection revealed that biomass of heterotrophic bacteria remained well under 1% of total biovolume.

Before starting the experiments, the inoculum was acclimatized for 10–15 days at different combinations of light intensity and temperature. Six light intensities (10, 40, 60, 100, 150, and 500 μmol of photons m^−2^ s^−1^) and four water temperatures (15, 20, 25, and 30 °C) were established. The light intensity was provided by daylight fluorescent lamps in a 12–12 h light-dark cycle. The flasks were agitated twice a day. The water temperatures tested were chosen based on the annual mean (25 °C), winter mean (20 °C), and the extreme temperatures (15 °C and 30 °C) observed in Camorim reservoir.

The combined effects of light and temperature on the *C. raciborskii* strains were studied in batch culture systems in triplicates. The experiments were setup in Erlenmeyer flasks of 300 mL, with 200 mL modified WC culture medium [35]. *C. raciborskii* strains were inoculated with initial biomass of 100 μg L^−1^ chlorophyll-*a* (Chl-*a*). The flasks were placed in incubators (SOLAB SL-224) under the 24 combinations of light and temperature and the growth was monitored for 10 days. Samples were collected daily for measurements of the Chl-*a* concentration and photosystem II efficiency (ϕPSII), analyzed with the phytoplankton analyzer Phyto-Pam (Heinz WalzGmbH, Effeltrich, Germany). The Phyto-PAM was calibrated against a spectrophotometric determination of Chl-*a* from the *C. raciborskii* strains, which was done with a 90% acetone extraction, based on Ritchie (2006) [36]. Phyto-Pam is a methodology that analyzes cells in vivo from fluorescence, with fluorescence being considered a rapid method for growth monitoring [37].

Growth rates (*μ*) were calculated from the chlorophyll-*a* increase using a logistic curve model (Equation (1)) that was fitted iteratively in SigmaPlot 12.5^®^ software.

(1) $$N_{t}=\frac{N_{0}*k}{{\left(N_{0}+(k-N_{0}\right)}^{*}\exp\left(-\mu *t\right))}$$

where *N_t_* = chlorophyll-*a*; *t* = time (days); *N*_0_ = initial value of chlorophyll-*a*; *k* = support capacity; *μ* = intrinsic rate of population increase (day^−1^).

STXs were analyzed in samples taken at the end of the experiment (day 10). Depending on cell density, 5–15 mL culture volume was filtered through a 25 mm diameter glass fiber filter (GF1, Sartorius, pore size: 0.7 µm) and filters were immediately stored in a freezer (−20 °C) until analysis. For extraction of PSP, the filters were soaked with 2 mL of acetic acid 30 mM and, for 1 min, sonicated at 30% of potency in falcon tubes placed in an ice bath. After this step, the samples were centrifuged at 4 °C for 15 min. The supernatant was collected, filtered through 0.22 µm PVDF filters (13 mm diameter—Analitica) and transferred into HPLC vials for subsequent analysis according to Diener et al. 2006 and 2007 [38,39]. STXs were analyzed using a post-column oxidation method with fluorescence detection (HPLC-FD), according to Diener et al. 2006 and 2007 [38,39] based on hydrophilic interaction liquid chromatography coupled fluorescence detector. Briefly, STX and its analogues were separated on a Shimadzu Prominence (Kyoto, Japan) liquid chromatography system equipped with a post-column reaction oven and a fluorescence detector (RF10AX). Commercially available standards of STX derivatives (National Research Council/Institute for Marine Biosciences, Halifax, NS, Canada) were employed in HPLC-FD experiments for compound identification. Toxins were detected using a fluorometric detector, with excitation at 330 nm and emission at 390 nm. Toxins were identified and quantified by comparison with known retention time and integrated areas of analytical standards. Calibration standards for all analyzed toxins (STX, dcSTX, NEO, GTX-2,3 dcGTX-2,3) were obtained from the Institute of Marine Bioscience, National Research Council of Canada (Halifax, NS, Canada).

The concentrations of gonyatoxins GTX2 + GTX3 (only GTX 2 and GTX3 were detected) were used to assess the cellular quota. The cell quota of STXs was determined by dividing the STXs concentration (µg mL^−1^) by cell density. Cell density was calculated from the sample counts of the last day (t10) fixed in lugol 2% in a Neubauer chamber through the optical microscope (Nikon Eclipse E-200 LED MV R, Nikon Corporation, Tokyo, Japan).

### Statistical Analysis

In order to verify the combined effect of light and temperature on the growth, production of STXs and STXs cellular quota in the three strains of *Cylindrospermopsis raciborskii*, two-way ANOVA with temperature and light as fixed factors were run. Prior to analysis, normality was tested using a Shapiro-Wilk test, whereas homogeneity of variance was tested by Levene’s Equal Variance Test. When normality and variance failed, the data were log-transformed. To detect differences between groups, Holm-Sidak post-hoc comparisons were carried out. In all analyses, the level of significance was set at *p* < 0.05. All statistical tests were performed using the SigmaPlot^®^ program version 12.5.

## Figures and Tables

**Table 1:** Two-way ANOVA table for effects of temperature and light intensity on growth rates of *C. raciborskii* strains.

Source of Variation	DF	F	*P*
CYLCAM-01			
Light	5	15.93	<0.001
Temperature	3	270.40	<0.001
Light × Temperature	15	5.18	<0.001
Residual	48		
Total	71		
CYLCAM-02			
Light	5	22.84	<0.001
Temperature	3	116.17	<0.001
Light × Temperature	15	11.18	<0.001
Residual	48		
Total	71		
CYLCAM-03			
Light	5	29.68	<0.001
Temperature	3	195.63	<0.001
Light × Temperature	15	13.72	<0.001
Residual	48		
Total	71		

DF = Degree of freedom; F = statistic values; *P* = significance level.

**Table 2:** Two-way ANOVA table for effects of temperature and light intensity on saxitoxin (STX) production of *C. raciborskii* strains.

Source of Variation	DF	F	*P*
CYLCAM-01			
Light	5	5.583	<0.001
Temperature	3	27.787	<0.001
Light × Temperature	15	5.292	<0.001
Residual	48		
Total	71		
CYLCAM-02			
Light	5	2.546	<0.041
Temperature	3	24.874	<0.001
Light × Temperature	15	5.162	<0.001
Residual	46		
Total	69		
CYLCAM-03			
Light	5	0.726	0.607
Temperature	3	7.644	<0.001
Light × Temperature	15	2.798	0.004
Residual	47		
Total	70		

DF = Degree of freedom; F = statistic values; *P* = significance level.

**Table 3:** Two-way ANOVA table for effects of temperature and light intensity on STXs cellular quota of *C. raciborskii* strains.

Source of Variation	DF	F	*P*
CYLCAM-01			
Light	5	1.42	0.232
Temperature	3	18.03	<0.001
Light × Temperature	15	3.58	<0.001
Residual	48		
Total	71		
CYLCAM-02			
Light	5	8.70	<0.001
Temperature	3	19.87	<0.001
Light × Temperature	15	6.87	<0.001
Residual	48		
Total	71		
CYLCAM-03			
Light	5	1.83	0.123
Temperature	3	2.35	0.083
Light × Temperature	15	1.82	0.058
Residual	48		
Total	71		

DF = Degree of freedom; F = statistic values; *P* = significance level.

## Supplementary Materials

The following are available online at http://www.mdpi.com/2072-6651/11/1/38/s1, Figure S1: Contribution of two saxitoxins variants (STXs) in 24 treatments combining different light and temperature conditions in three *C. raciborskii* strains. CYLCAM-01 (**A**), CYLCAM-02 (**B**) and CYLCAM-03 (**C**). Light intensities (µmol of photons m^−2^ s^−1^) = 10, 40, 60, 100, 150 and 500. Temperatures = 15, 20, 25 and 30 °C.
