# Supplementary material for: Combined Effect of Light and Temperature on the Production of Saxitoxins in Cylindrospermopsis raciborskii Strains
Source: Toxins (Basel). 2019 Jan 14;11(1):38. doi: 10.3390/toxins11010038 (PMC6356899; doi:10.3390/toxins11010038)
Supplement: Supplementary file 1 [file toxins-11-00038-s001.pdf]

Supplementary Materials: Combined Effect of Light and Temperature on the Production of Saxitoxins in *Cylindrospermopsis raciborskii* Strains

Marcella C. B. Mesquita, Miquel Lürling, Fabiane Dorr, Ernani Pinto, and Marcelo M. Marinho

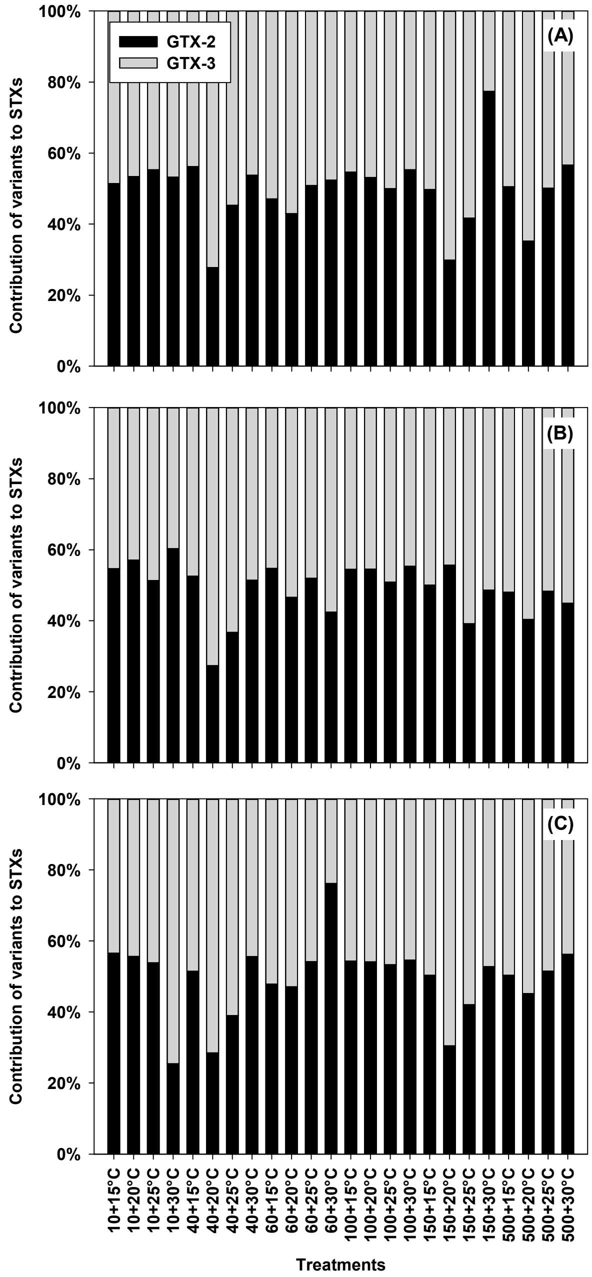

Figure S1. Contribution of two saxitoxins variants (STXs) in 24 treatments combining different light and temperature conditions in three *C. raciborskii* strains. CYLCAM-01 (**A**), CYLCAM-02 (**B**) and CYLCAM-03 (**C**). Light intensities (μmol of photons m^{-2} s^{-1}) = 10, 40, 60, 100, 150 and 500. Temperatures = 15, 20, 25 and 30 °C.
